# Polyphenolic Nutrients in Cancer Chemoprevention and Metastasis: Role of the Epithelial-to-Mesenchymal (EMT) Pathway

**DOI:** 10.3390/nu9080911

**Published:** 2017-08-21

**Authors:** Haneen Amawi, Charles R. Ashby, Temesgen Samuel, Ramalingam Peraman, Amit K. Tiwari

**Affiliations:** 1 Department of Pharmacology and Experimental Therapeutics, College of Pharmacy and Pharmaceutical Sciences, University of Toledo, Toledo, OH 43614, USA; haneen.amawi@rockets.utoledo.edu; 2 Pharmaceutical Sciences, College of Pharmacy, St. John’s University Queens, New York, NY 11432, USA; cnsratdoc@optonline.net; 3 Department of Pathology, School of Veterinary Medicine, Tuskegee University, Tuskegee, AL 36088, USA; tsamuel@mytu.tuskegee.edu; 4 Medicinal chemistry Division, Raghavendra Institute of Pharmaceutical education and Research (RIPER)-Autonomous, Anantapur 515721, India; drramalingamp@gmail.com

**Keywords:** polyphenols, chemoprevention, cancer, metastasis, epithelial mesenchymal transition

## Abstract

The epithelial-to-mesenchymal transition (EMT) has received significant interest as a novel target in cancer prevention, metastasis, and resistance. The conversion of cells from an epithelial, adhesive state to a mesenchymal, motile state is one of the key events in the development of cancer metastasis. Polyphenols have been reported to be efficacious in the prevention of cancer and reversing cancer progression. Recently, the antimetastatic efficacy of polyphenols has been reported, thereby expanding the potential use of these compounds beyond chemoprevention. Polyphenols may affect EMT pathways, which are involved in cancer metastasis; for example, polyphenols increase the levels of epithelial markers, but downregulate the mesenchymal markers. Polyphenols also alter the level of expression and functionality of important proteins in other signaling pathways that control cellular mesenchymal characteristics. However, the specific proteins that are directly affected by polyphenols in these signaling pathways remain to be elucidated. The aim of this review is to analyze current evidence regarding the role of polyphenols in attenuating EMT-mediated cancer progression and metastasis. We also discuss the role of the most important polyphenol subclasses and members of the polyphenols in reversing metastasis and targeting EMT. Finally, limitations and future directions to improve our understanding in this field are discussed.

## 1. Introduction

The consumption of fruits and vegetables rich in certain polyphenols has been reported to decrease the incidence and mortality of cancer, as well as delaying cancer progression [1]. The adverse effects and toxicities of many of the currently chemotherapeutic drugs have spurred research with certain phytochemicals for chemoprevention and treatment of cancer [2,3]. Polyphenols are natural phytochemicals that are present in high amounts in many plants (including plant seasonings), fruits, vegetables, seeds, oils, and alcoholic and non-alcoholic beverages [4]. Indeed, polyphenols have been shown to inhibit the proliferation of cancers of the mouth, gastrointestinal tract, liver, lung, breast and skin both *in vivo* and *in vitro* [5]. However, the molecular mechanisms that mediate the chemopreventive efficacy of the polyphenols remain to be elucidated to a large extent. Furthermore, there are considerable discrepancies between the health benefits versus the clinical outcomes with the intake of polyphenol. This could be due to a number of factors, with one of the major ones being the testing of non-physiological concentrations of polyphenols, thereby potentially obscuring the mechanism of action at therapeutic doses. Current meta-analyses of observational studies showed limited evidence of a correlation between dietary polyphenol intake and cancer risk, with most of the significant findings related to a decreased risk of lung, stomach, breast, and colorectal cancers [6]. Nonetheless, accumulating evidence suggests that epithelial-mesenchymal transition (EMT), one of the main pathways involved in cancer development and metastasis, is modulated by a number of polyphenolic compounds [7,8]. During EMT, cells transition from an epithelial to a mesenchymal state and lose their cell–cell adhesions, cell polarity and differentiation properties [9]. These changes induce the cells to become motile and invasive, allowing migration through the extracellular matrix, reaching distant tissues [10]. Numerous polyphenolic compounds, including, but not limited to, flavonoids, ellagic acid, quercetin, silymarins, resveratrol and curcumin, have been reported to significantly reverse metastasis and invasiveness in different cancers *in vitro* and *in vivo*. Therefore, it is possible that polyphenolic compounds may reverse or prevent cancer progression, invasion and metastasis by inhibiting the EMT signaling pathways in cancer cells. However, most of the reported benefits of polyphenols were obtained from preclinical studies in cells or animals where the doses used were significantly higher than the amount usually obtained from the consumption of food [11]. The exact intake and bioavailability of polyphenols are still unknown, greatly restricting their clinical use for cancer chemoprevention, treatment and reversal of metastasis.

To the best of our knowledge, only a few previous comprehensive reviews have been published describing the effects of different phytochemicals on EMT and metastasis [7,8]. Our review specifically describes the effects of polyphenols on EMT signaling and related proteins in preventing and treating cancer metastasis. The goal of this review is to provide the reader with a better understanding of the interactions between natural polyphenols and the inhibition of EMT signaling and chemoprevention for cancer development and metastasis. This review will present some of the complex molecular mechanisms that may be involved in mediating the chemo-preventative efficacy of the natural polyphenols.

## 2. Dietary Polyphenols and Health Benefits

**Figure 1:**
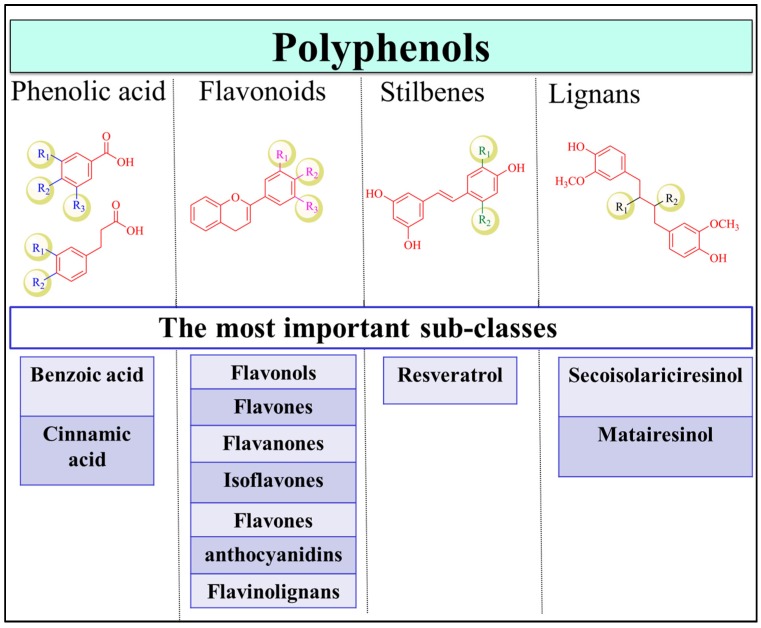
Polyphenol classification. The classes of polyphenols include phenolic acids, flavonoids, stilbenes, and lignans. Examples of important subfamilies of each class are shown.

Polyphenols are one of the most abundant phytochemicals in plants and have the highest intake by humans compared to other natural products [12]. Fruits, vegetables, herbs, cereals, tea, coffee, nuts, seeds and beer are rich sources of polyphenols [13]. Polyphenolic compounds are secondary metabolites that accumulate in the plant leaves and flowers to protect plants against diseases, infections and damage [14,15,16]. The structures of the family members are highly diverse and complex, with molecular weights ranging between 500 and 3000 Da [17]. Therefore, depending on the number of phenolic rings and the interconnection of these rings, the polyphenolic family is divided into several major classes, with thousands of members in each class, including flavonoids, phenolic acids, stilbenes, and lignans (Figure 1) [13]. Coumarins are also considered as a separate subgroup of polyphenols that range from simple to polycyclic coumarins [18,19]. Flavonoids are the largest class in the group and can be categorized as flavonols (e.g., quercetin, kaempferol) [20], flavones (e.g., apigenin, luteolin) [21], flavanones (e.g., naringenin and hesperetin) [22], isoflavones (e.g., genistein and daidzein) [23,24], flavanols (e.g., catechin, epigallocatechinepigallocatechin gallate) [18,25], anthocyanins (e.g., cyandin, malvidin) [26], and flavonolignans. Phenolic acids are the second most common class of polyphenols after flavonoids and are present in coffee and black tea [27]. They are primarily classified as benzoic and cinnamic acid derivatives [28]. Stilbenes are not common in plants and are only produced upon pathogen invasion [29]. Finally, lignans are phytoestrogens that are highly abundant in flaxseed and flaxseed oil, with the best-known compounds being secoisolariciresinol and matairesinol [30]. The structure of polyphenols is an important determinant of their bioavailability, pharmacokinetic profile, interactions with biomolecules and efficacy [30].

Polyphenols are reducing compounds that have been reported to prevent inflammation [31], oxidative stress [12], cardiovascular disease [32], infections [13,33], bone diseases [34] and cancer [35], among others [36]. They can form complexes with certain proteins, scavenge free radicals and modulate several signaling pathways [37,38]. However, the diversity of the distribution of dietary polyphenols in plants, complexation with other plant constituents, degradation of these compounds during food processing, and their poor pharmacokinetic profile due to their complex structures and interactions with gut microflora and other biomolecules limit the efficacy of polyphenols [30]. In addition, the exact determination of the daily intake of dietary polyphenols is still uncertain and is subject to significant variation between different individuals. Moreover, the diversity of the structures in this family makes bioavailability a function of the individual polyphenols. Generally, after oral ingestion, polyphenols are present in low concentration in the blood and urine, as they interact with gut microflora, are primarily metabolized by methylation, glucuronidation and sulfation, and are rapidly excreted in the bile and urine [39]. However, the metabolites of certain polyphenolic compounds can significantly contribute to the biological activity or efficacy of the parent polyphenolic compounds [40]. Clearly, additional research is required to improve our knowledge about the intake and bioavailability of dietary polyphenols to determine their efficacy in disease prevention.

### Polyphenols in Cancer Prevention and Treatment

Cancer is an amalgam of complex, heterogeneous diseases, in which many signaling pathways are affected [41]. The processes of initiation, promotion and progression are required for the development and invasiveness of cancer [42]. In cancer cells, there is a significant disruption of the cell cycle, uncontrolled cell proliferation, and dysregulation of processes that produce cell death, such as apoptosis [43]. Dietary factors, especially those from plants, have been reported to reduce the risk of various cancers and malignancies [44]. According to the American Cancer Society (cancer facts and figures, 2017), about 20% of cancers in the United States can be prevented by a healthy lifestyle, including a diet rich in vegetable and fruits and physical activities [45]. Polyphenol-rich foods, based on preclinical, clinical and epidemiological studies, have been reported to have chemopreventive and anticancer efficacy [46,47,48]. Polyphenolic compounds can inhibit the proliferation of prostate, bladder, lung, gastrointestinal, breast and ovarian cancers [49]. Quercetin, resveratrol, green tea polyphenols [50], epigallocatechin-3-gallate [51] and curcumin [52] have efficacy as anticancer compounds. It has been hypothesized that polyphenols may prevent (1) cancer initiation (cytoprotective); (2) relapse; or (3) its progression and metastasis to distant organs (cytotoxic) [53,54,55]. The cytoprotective (i.e., chemopreventive) effect of the polyphenols is attributed primarily to their antioxidant activities [56]. However, the actual anticancer efficacies of polyphenols are due to antioxidant-independent mechanisms, including their pro-oxidant action [49,56]. Therefore, polyphenols may produce antioxidant effects in normal cells, while inducing pro-oxidant damage in cancer cells.

Previously, it was postulated that the chemopreventive efficacy of polyphenols is primarily due to their antioxidant action [5]. Polyphenolic compounds have hydroxyl groups that donate their protons to a reactive oxygen species (ROS) [44]. In addition to their antioxidant effects, polyphenols inhibit the activity of phase I enzymes, primarily cytochrome P450 enzymes (CYPs), such as CYP1A1 and CYP1B1 [57]. This prevents the formation of reactive and carcinogenic metabolites [57]. The polyphenols also induce phase II enzymes, which increase the formation of polar metabolites that are readily excreted from body [58]; for example, dietary polyphenols inhibit the development of lung cancer and exert significant chemopreventive effects *in vitro* and *in vivo* [44]. Furthermore, it has been reported that certain dietary polyphenols decrease the cellular formation of ROS, which decreases the oxidation of DNA, proteins and lipids [44].

However, recent data suggests that the pro-oxidant, not the antioxidant, properties of the polyphenols may be important in treating and preventing cancer. The pro-oxidant activities of polyphenols in cancer cells generates ROS, producing [49,59,60] (1) cell cycle arrest [61]; (2) induction of apoptosis and DNA fragmentation [62]; (3) inhibition of proliferation signaling pathways, including epidermal growth factor receptor/mitogen activated protein kinase (EGFR/MAPK), phosphatidylinositide 3-kinases/protein kinase B (PI3K/Akt) [63], and nuclear factor kappa-light-chain-enhancer of activated B cells (NF-ĸB) [64] and (4) anti-inflammatory effects [65,66]. For example, the polyphenols, at 0.3 mg/ml, from apples, inhibit human bladder transitional cell carcinoma (TCC, TSGH-8301 cells) proliferation, induce G2/M cell cycle arrest, and promote apoptosis and mitotic catastrophe [67]. Green tea polyphenols (e.g., pigallocatechin gallate and black tea theaflavins), in human papilloma virus-18-positive HeLa cervical cancer cells induced death, cell cycle arrest at the subG1 phase, apoptosis through caspases activation, and ROS generation. These aforementioned effects were mediated by inhibiting Akt and NF-κB signaling in these cells [68].

Flavonoids (e.g., apigenin, quercetin, luteolin, fisetin) were reported to induce apoptosis in cancer cells, including leukemic U937 cells [69], prostate cancer cells [70], hepatic cancer cells [71], and others. Curcumin is a polyphenol extracted from *Curcuma longa* (tumeric) [72]. Its anticancer efficacy was shown to be mediated through different mechanisms, including TNF-induced apoptosis, the inhibition of NF-κB, and the inhibition of Wnt/β-catenin and EMT signaling in breast, colon, brain and other cancers [73]. However, as mentioned earlier, the efficacy of the polyphenols is limited by their less than optimal pharmacokinetic profile [74]. Certain polyphenols lack significant efficacy due to their limited bioavailability, the amount consumed and the cancer type and stage [75,76]. Accordingly, the diversity in the response to polyphenols is dependent upon the dose, cancer cell type, and the patient’s genome [42].

## 3. Polyphenols Role in Reversing EMT Mediated Cancer Metastasis

### 3.1. Metastasis

The metastasis of cancer consists of at least five phases and ultimately >90% of relapsed cancer patients die from metastasis [77]. A significant proportion (at least 70%) of cancer patients has metastasis following their initial cancer diagnosis [78]. In metastasis, the cancer cells degrade the basement membrane, invade the surrounding vasculature, enter into the blood and/or lymph circulation, migrate to the distant tissues and organs, and finally implant, colonize and proliferate to form new tumor masses [79]. The proposed molecular mechanisms involved in the development of cancer metastasis are very complex and numerous, involving different, unrelated genes [80,81]. This complexity interferes with the prediction of the probability of the development of metastasis, which affects the diagnosis and treatment of metastatic disease [78]. The tumor cells adopt new phenotypes, characterized by the overexpression of certain genes associated with metastasis, in combination with the downregulation of genes that when transcribed, produce proteins that inhibit metastasis [82]. Numerous studies have reported the differential expression of genes during metastasis, where numerous signaling pathways, including EMT, are dysregulated [83,84,85,86,87]. The level of metastasis is dependent upon a number of variables, including, but not limited to, the type of cancer, cancer stage, patient genetic profile and mutations and gender [88,89]. Currently, there is no clinically approved treatment that specifically targets metastasis. Typically, metastasis is treated using conventional therapies, such as radiotherapy, chemotherapy and surgery, which are used to treat primary tumors. The goal of the treatment for metastatic cancer is to control the metastasis and improve patient survival, as opposed to curing the disease [90]. Thus, it is imperative to find treatments that target molecules involved in the development and maintenance of metastasis.

### 3.2. EMT Role in Mediating Cancer Metastasis

**Figure 2:**
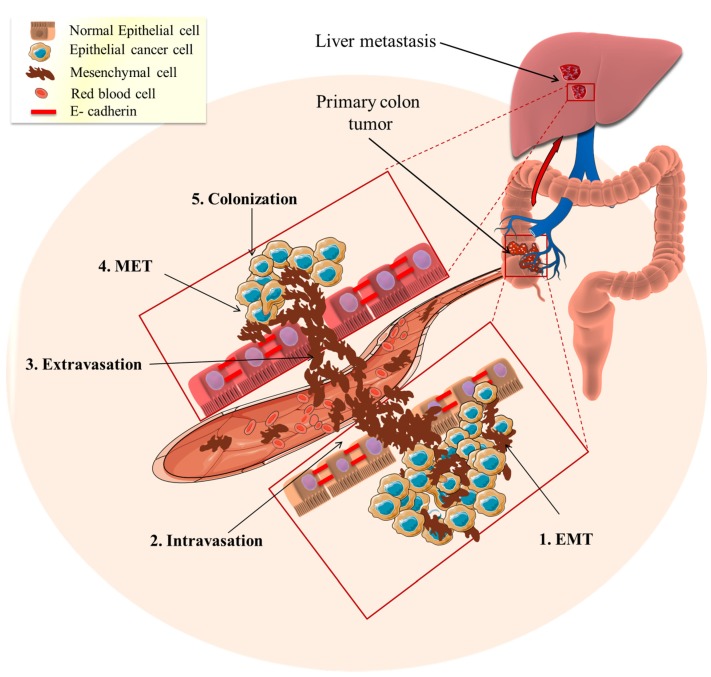
Epithelial to mesenchymal transition (EMT) role in cancer metastasis. The tumor epithelial cells transform mesenchymal invasive cells through EMT (1. EMT). Subsequently, the mesenchymal cells enter the blood circulation to distant places (2. Intravasation) and this results in the homing of circulating tumor cells to specific organs or tissues (3. Extravasation). The metastasized mesenchymal cells transition to the epithelial phenotype through the mesenchymal–epithelial transition (4. MET). The MET-transformed cancer cells become implanted and interact to form new colonies (5. Colonization) and ultimately forming a tumor.

Although EMT is essential for the developmental formation of organs and tissue repair, its pathological activation can lead to an increased risk of cancer and fibrosis [9]. Accumulating data suggests that the epithelial-mesenchymal transition (EMT) is one of the primary and early pathways involved in cancer development and metastasis [10,91,92]. In the early stages of metastasis, EMT activates cells to transition from an epithelial to a mesenchymal state where cells lose their cell–cell adhesions, cell polarity and differentiation properties (Figure 2). These changes decrease the probability of apoptosis and increase cell motility and tissue invasiveness, allowing them to migrate through the extracellular matrix, reaching distant tissues (Figure 2) [10]. On a morphological level, the cells lose their round shape and become spindle-shaped, which is associated with an increase in mobility and invasiveness [92]. This occurs, in part, from the loss of epithelial cell adhesion proteins, (e.g., E-cadherin) and the upregulation of the expression of mesenchymal proteins (e.g., N-cadherin, vimentin) [93]. E-cadherin is a transmembrane glycoprotein that is typically highly expressed on differentiated epithelial cells, where it regulates cell adhesion [94]. The adhesion process involves the formation of interactions between E-cadherin, located at the surface of the neighbor cells [95].

**Figure 3:**
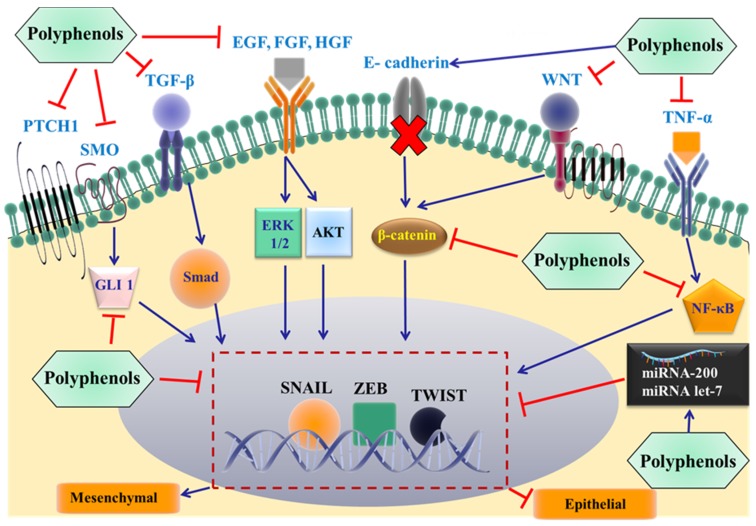
Proposed mechanisms by which dietary polyphenols inhibit EMT and cancer metastasis. The most important extracellular signals are shown, and these promote the epithelial to mesenchymal transition by binding to individual membrane receptors, eventually activating specific EMT-inducing transcription factors (snail, Zeb, and/or twist). The effect of the polyphenols on specific proteins in the signaling pathways at different levels remains to be elucidated. TGF-β: transforming growth factor β; EGF: epidermal growth factor; HGF: hepatocyte growth factor; FGF: fibroblast growth factor; PTCH1: Patched 1; SMO: smoothened; WNT: glycoprotein family; TNF-α: Tumor necrosis factor-α; NF-κB: nuclear factor kappa-light-chain-enhancer of activated B cells; GLI 1: glioma-associated oncogene 1; ERK: extracellular regulated protein kinases; AKT: protein kinase B.

The loss of E-cadherin expression is a hallmark and direct event required for the development of EMT, which is positively correlated to the development of metastatic cancers [94]. Alterations in various signaling pathways have been shown to induce the loss of E-cadherin and facilitate the transition from epithelial to mesenchymal state. For example, EMT can be regulated by extracellular signal-regulated kinases (ERK1/2) [96], tyrosine kinases [97], transforming growth factor β (TGFβ) [98], insulin like growth factor (ILGF) [99], epidermal growth factor (EGF) [100], platelet derived growth factor (PDGF) [101], NF-κB [102], protein kinase B (Akt) [103] and the (Wnt)/β-catenin signaling pathway (Figure 3) [104]. All the above proteins can activate nuclear transcriptional factors, such as snail, twist and zinc finger E-box binding homeobox (ZEB1/2), which directly bind to the E-cadherin gene promoter region, suppressing gene expression [105,106,107,108]. In addition, mutations in oncogenes and suppressor genes, DNA methylation and changes in microRNAs are also involved in the induction process [108]. In contrast to EMT, the mesenchymal–epithelial transition (MET) is the transition of mesenchymal cells to an epithelial phenotype [109]. The presence of EMT and MET provides cancer cells with flexibility, where initially they use EMT to detach, pass through the basement membrane and reach the distant tissues. Subsequently, the cells can adopt the epithelial phenotype again, leading to the development of metastasis [10].

Wnt/β-catenin signaling and EMT are closely related and are involved in the positive feedback activation of one another [110]. Wnt/β-catenin signaling is a crucial pathway in cell–cell communication, where one cell releases the Wnt protein to bind to an adjacent cell’s surface receptor [111]. This binding results in the initiation of cellular signals that are transmitted to the nucleus to induce genetic modifications [112]. β-catenin is the key regulatory protein for signal transmission in Wnt signaling pathways [111]. It is also involved in structural and cell adhesion functions, where it connects the membrane-associated E-cadherin to cellular actin [95]. Upon activation by Wnt, cytosolic β-catenin is stabilized and translocated from the cytoplasm to nucleus, interacting with DNA binding proteins, such as lymphocyte enhancer factor and T cell factor complex (TCF/EF1), inducing the transcription of several genes (e.g., T-cell factor 1 (TCF1), matrix metalloproteinase-7 (MMP-7), the cluster of differentiation 4 (CD4) protein and cyclin D1) [113]. Finally, these genes regulate cellular proliferation, development, survival and metastasis [114]. In the absence of the extracellular Wnt, β-catenin translocation to the nucleus is inhibited by the β-catenin destruction complex, which consists of adenomatous polyposis coli (APC), axin, glycogen synthase kinase (GSK3β) and casein kinase 1 (CK1α)) [115,116].

E-cadherin binds directly to β-catenin, where the conserved carboxyl terminus of E-cadherin binds to the APC binding site of β-catenin in the cytoplasm and interacts with actin microfilaments [117]. This binding is essential for mediating cadherin-based cell adhesion with cytoskeletal actin. A decrease in the expression of E-cadherin is positively correlated with an increased activation of Wnt/β-catenin signaling, induction of EMT, and rapid progression of cancer [118,119]. Another interaction between the two pathways involves the zinc finger transcriptional receptor, snail. It binds to the promoter region of the E-cadherin gene, mediating the repression of E-cadherin gene expression [120]. The repression of E-cadherin is positively correlated with the upregulation of Wnt/β-catenin signaling [121]. Snail and β-catenin interact physically and functionally, exerting a positive feedback effect on one another [122]. The pooling or accumulation of snail in the cytoplasm is regulated by the GSK3β-induced phosphorylation of snail [122,123]. Thus, Wnt signaling can stabilize and increase the levels of snail in the cytoplasm by the inhibition of GSK3β [123]. Both snail and β-catenin synergize and stabilize each other’s biological effects and transcriptionally augment E-cadherin-induced repression on epithelial cells and facilitate EMT in cancer [124].

Recently, EMT has been identified and recognized as one of the factors contributing to the development of multidrug resistance (MDR) in cancer cells [125]. Several studies indicate that different cancer cells expressing MDR phenotypes also typically express the EMT phenotype [126]. These MDR cells express higher levels of N-cadherin and vimentin, but express lower levels of E-cadherin and cell-cell junctions, suggesting that EMT is positively correlated with the MDR phenotype [125,127]. In addition, one study has shown that cells undergoing EMT have a significantly lower response to the chemotherapeutic drug, cyclophosphamide, compared to cells not undergoing EMT [128]. Consequently, MDR could be targeted by developing drugs that also inhibit pathologically-induced EMT [127,128]. Finally, inhibiting EMT could be a promising strategy to limit cancer cell diffusion and stemness, which could lower the rate of mortality in patients with metastatic cancer.

### 3.3. Polyphenols Effectiveness in Reversing Metastasis

The initial studies with polyphenols were predominantly focused on reducing cancer risk by dietary polyphenols, as well as the treatment of primary tumors [129]. However, over the past decade, there has been a significant increase in determining the efficacy of polyphenols in metastatic cancer [130]. Indeed, dietary polyphenols, such as epicatechin, epigallocatechin (EGC), delphinidin tannins, epigallocatechin-3-gallate (EGCG), green tea catechins, quercetin and luteolin among others, inhibit cell wound healing and transwell migration *in vitro* in several cancer cell lines [131,132]. Carnosol, a dietary diterpene, has been reported to inhibit the viability of human breast, ovarian and intestinal tumor cell lines [133]. The combination of carnosol with curcumin also significantly inhibited the viability of primary cancer cells isolated from the pleural fluid or ascites of patients with metastatic cancers [133]. The antimetastatic efficacy of certain polyphenols was also reported *in vivo*, where polyphenols inhibited the development of metastasis and significantly improved the survival of animals in metastatic models [134]. The mechanisms by which these compounds produce antimetastatic efficacy are complex. For example, polyphenols significantly downregulate the expression of matrix metalloproteinases, such as MMP-2 and MMP-9, which promote cellular invasion [135,136]. The hepatocyte growth factor receptor (HGFR or MET) activates epithelial cell dissociation and invasive branching, is also significantly inhibited by several polyphenols, including (−)-Epigallocatechin-3-gallate (EGCG) [137,138]. Polyphenols significantly reduce the activation and the nuclear translocation of NF-κB, further inhibiting the signaling pathways and affecting certain genes [139,140]. The formation of new blood vessels, known as angiogenesis, is considered to be a primary mechanism for metastatic cells to reach the blood and migrate to new sites in the body [141]. Polyphenols have been reported to interfere with vascular endothelial growth factor (VEGF)-mediated angiogenesis [142]. Finally, polyphenols also decrease EMT signaling and reverse metastasis by altering the levels of proteins involved in this pathway.

### 3.4. Polyphenols Reverse Metastasis by Targeting EMT

As discussed above, polyphenols, in part, produce their antimetastatic efficacy by targeting the EMT pathways. Currently, only a few studies have determined the effects of whole plant polyphenols extract. The majority of the studies were focused on a certain class or specific polyphenolic compounds. Consequently, this does not allow researchers to determine the effect of whole plant polyphenol extracts on EMT and related pathways. There are data suggesting that whole polyphenol extracts can reverse metastasis and modulate EMT. For example, the extracted polyphenols from *A. annua* L. (pKAL), including hydroxycinnamic acids and flavonoids, were evaluated in metastatic breast cancer cells (MDAMB-231) [143]. The compound pKAL significantly inhibited the TNF-α-induced migration and invasion of MDAMB-231 cells (1–30 µg/mL), with minimal toxicity in normal cells. Furthermore, pKAL caused cells to become a more rounded, epithelial-like phenotype [143]. pKAL also significantly inhibited the mesenchymal markers N-cadherin and snail, but not E-cadherin or β-catenin. Thus, the modulation of EMT could play a role in the anti-metastatic mechanism of action in MDAMB-231 cells [143]. In addition, the anti-metastatic efficacy of the polyphenol mixtures, mainly quercetin and kaempferol from *Euphorbia supina* (PES), was determined in metastatic breast cancer MDA-MB-231 cells [46]. PES significantly inhibited the expression levels of N-cadherin, snail and MMP-9 at concentrations up to 5 µg/mL [144]. Curcumin (20–40 µM) significantly inhibited the Wnt/β-catenin-mediated activation of EMT, where β-catenin, vimentin, and TCF4 were significantly downregulated and E-cadherin was significantly upregulated in SW620 cells [145]. These findings support the hypothesis that polyphenols may produce their chemopreventive and antimetastatic efficacy primarily by modulating EMT and its network of proteins. The role of the most important classes of polyphenols and their members in the prevention and reversal of EMT-mediated cancer metastasis is discussed below in detail.

#### 3.4.1. Flavonoids

Flavonoids are the most abundant dietary polyphenols in fruits, vegetables, flowers, chocolate, tea, wine and other edible plants [74]. The basic chemical structure for all members in the flavonoid family is two benzene rings, connected by a 3-carbon bridge, forming a heterocycle (i.e., C6–C3–C6) [146]. The anticancer and antimetastatic efficacy of the flavonoids has been investigated extensively and confirmed in both *in vitro* and *in vivo* models [147]. However, the exact mechanisms by which these compounds produce their efficacy remains to be elucidated, as with other polyphenols [148]. However, there is robust evidence that modulation of EMT and its related signaling pathways is one of the primary determinants of their efficacy [149]. For example, epigallocathechin gallate (EGCG), a flavan-3-ol, induced apoptosis (40 µM), significantly inhibited colony formation and cell migration (20 and 40 µM) in nasopharyngeal carcinoma (NPC) cancer stem cells (CSC), including the sphere-derived NPC TW01 and TW06 cell lines [150]. Interestingly, EGCG significantly upregulated the expression of the epithelial marker, E-cadherin, and downregulated the expression of snail and vimentin at 20 and 40 µM [150]. The incubation of the invasive and metastatic A431-III cell line with 20 µM luteolin and quercetin, for 48 h, significantly suppressed the expression of the following mesenchymal markers and transcriptional factors: Fibronectin, vimentin, twist, snail, and N-cadherin and relocalized E-cadherin adhesions on the cell membrane, yielding a more epithelial-like phenotype [151]. In addition, 20 µM of luteolin and quercetin reversed the migration and invasiveness of the A431-III cells and downregulated MMP-9 expression, which significantly induces EMT and cell invasion [151].

#### 3.4.2. Stilbenes

The stilbene polyphenolic compounds are characterized by a di-methylene bridge connecting the two phenolic rings [152]. Resveratrol is the most well-known of stilbenes and has been reported to significantly inhibit the metastasis of various cancers [136,153,154]. Resveratrol significantly inhibits the metastasis of colon cancer cell line (LOVO) both *in vitro* and *in vivo* (significantly at >50 mg/kg) [153]. Resveratrol also significantly inhibited gastric cancer cell (SGC-7901) metastasis *in vitro* and reversed both hedgehog and EMT signaling at ≈100 µM [155]. The glioma-associated oncogene 1 (Gli-1), N-cadherin and snail were significantly downregulated, in tandem with a subsequent significant upregulation in E-cadherin levels [155]. Resveratrol (6–60 µM) induced the presence of epithelial characteristics in cancer cells following their exposure to compounds that induce EMT [154,156]. For example, resveratrol, at 6 μM and 12 μM, significantly inhibited TGF-β1-induced (10 ng/mL for 48 h) EMT in LOVO cells by upregulation of E-cadherin and downregulation of vimentin [153]. Similarly, epidermal growth factor (100 ng/ml) induced EMT in MCF-7 breast cancer cells, and this was prevented and reversed by 25 µM of resveratrol, which, in turn, inhibited EMT [154]. Resveratrol, at 25 μM, inhibited wound healing and cell motility, significantly downregulated vimentin and N-cadherin, and significantly upregulated E-cadherin levels in MCF-7 cells [154]. Resveratrol (25 µM) also significantly downregulated EMT-inducing transcriptional factors, such as ZEB 1/2 and snail [154]. Similar results were obtained in ovarian cancer cells, where resveratrol (20–60 µM) significantly inhibited cisplatin-induced EMT [156]. Finally, novel analogues of resveratrol have been reported to enhance the inhibition of the proliferation and migration of ovarian cancer cells, compared to resveratrol [157]. These analogues also inhibited the expression of epithelial to mesenchymal transition (EMT) markers [157]. Overall, these results indicate that resveratrol exerts a significant part of its anti-metastatic efficacy through altering the EMT pathway.

#### 3.4.3. Phenolic Acids

The phenolic acid class of polyphenols is the second most abundant group in the family, accounting for 30% of the dietary polyphenols [158]. The phenolic compounds are divided into either hydroxybenzoic acid compounds, such as gallic acid or hydroxycinnamic acid, such as caffeic acid and ferulic acid [159,160]. These members of the phenolic family were evaluated for anticancer and antimetastatic efficacy and their effects on the mesenchymal characteristics of cancer cells [161,162]. Caffeic acid, ellagic acid, gallic acid and ferulic acid inhibit cancer cell proliferation and metastasis in different cancer models [161,163,164,165]. Furthermore, anacardic acid suppresses prostate cancer angiogenesis and migration alone or in combination with radiation by different mechanisms [166,167]. In primary human umbilical vascular endothelial cells (HUVECs), anacardic acid (20 µM) inhibited Src and focal adhesion kinase (FAK), activation of RhoA-GTPase and inactivation of Rac1 and Cdc42-GTPases [167]. Phenolic compounds have also been reported to reverse metastasis induced by EMT. For example, caffeic acid (50–100 µM for 48 h) significantly inhibited the migratory capability and reversed EMT to MET (epithelial and adhesive phenotype) in the skin cancer cell line, HaCaT [168]. Additionally, caffeic acid (100 μM) significantly inhibited the activation of the NF-κB/snail signal pathway, a strong inducer of EMT [168]. Furthermore, E-cadherin expression was significantly increased, whereas N-cadherin and vimentin levels were significantly downregulated by caffeic acid (100 µM) [168]. Ferulic acid significantly inhibits EMT-induced metastasis of MDAMB-231 cells *in vitro* (10–30 µM) and in an *in vivo* mouse xenograft model (100 mg/kg/day) [165]. Ferulic acid (200 µM) significantly blocked P-Smad2/3 activation and attenuated all of the EMT changes induced by 5 ng/ml of TGF-β1 in rat kidney epithelial cells (NRK-52E) [169]. Furthermore, ferulic acid (200 µM) significantly upregulated E-cadherin and downregulated fibronectin, snail, and integrin linked kinase (ILK) in the same cell line [169]. Although the direct effect of other phenolic compounds was not investigated directly on EMT markers, such as N-cadherin or vimentin, they modulated other proteins that significantly affect EMT. Proteins, such as MMP-9, TGF-β, IL-6, β-catenin, NFκB, VEGF, and other oncogenic signaling proteins, were significantly downregulated by caffeic acid, ellagic acid, gallic acid and other phenolic acid compounds at 50–200 µM [66,161,169,170,171,172]. Ellagic acid, when combined with luteolin and punicic acid, at 64 µg/component/day, significantly inhibited primary prostate cancer growth, prevented metastasis, and significantly decreased VEGF and IL6 levels *in vivo* [172]. Gallic acid significantly inhibited the migration and invasion of A375.s2 human melanoma cells by inhibiting the zinc-dependent, proteolytic enzyme MMP-2 [173].

#### 3.4.4. Lignans

The dimerization of two cinnamic acids results in a 2,3-dibenzylbutane structure, forming the lignans class in the polyphenolic family [174]. Secoisolariciresinol and matairesinol are the two most well-known lignans [175]. Lignans have been shown to have chemopreventitive efficacy in breast and prostate cancers in humans [176,177,178]. Flaxseed supplementation of secoisolariciresinol (SDG) at 73, 147 or 293 μmol/kg, which is equivalent to 2.5%, 5% or 10% flaxseed diet, significantly reduced metastasis in a melanoma mice model where the percent of animals that had >50 lung tumors was reduced to 30%, 21%, and 22%, respectively, compared to the control (59%) [178]. In addition, a 10% flaxseed diet, given as 0.2 g/kg secoisolariciresinol diglycoside (SDG) and 36.53 g/kg flaxseed oil (FO), significantly reduced the growth and metastasis of estrogen receptor positive breast cancer, in part due to the presence of lignan [179]. However, to date, there have been no published reports correlating the antimetastatic potential of lignans to EMT modulation. Further studies are required to the effect of polyphenolic lignans on EMT and its interlinked pathways.

## 4. Future Directions

### 4.1. Effects of Polyphenols on Specific Proteins in the EMT Pathway

Currently, it is unknown as to what specific proteins in the EMT pathway or related pathways are affected by the polyphenols. It remains to be determined if polyphenols are altering the EMT pathways directly or act through the higher level signaling pathways or via a combination of multiple targets. A summary of the general effects of important polyphenols on EMT markers and related proteins are listed in Table 1. The direct effect of polyphenols on specific targets can be achieved by studying the EMT and related signaling pathways at the level of gene expression. For example, investigating the impact of dietary polyphenols on the expression levels and activities of microRNAs that control the mesenchymal properties of epithelial cells has not been thoroughly evaluated. Recently, the correlation of microRNA with cancer is gaining significant interest [176]. Such research would provide a better understanding and new perspectives in defining the exact targets for the efficacy of polyphenols and their exact relationship with EMT in cancer metastasis.

#### Studying the Impact of Polyphenols on EMT-Related miRNAs

MicroRNAs (miRNA) are a specific type of single stranded RNA sequences that do not code for proteins. These sequences are usually short in length, consisting of only a few nucleotides (typically 19–25) and they negatively regulate their targets [180,181]. miRNA act at the mRNA level, where they complementary interact with their target mRNA. However, their binding sites lack strict specificity, so each miRNA can bind and target several mRNA, affecting apoptosis [182], disease progression [183], cancer development and metastasis [184,185]. The miRNAs are initially expressed from their genes by RNA polymerase II as Pri-miRNA. Subsequently, they become either pre-miRNA in the nucleus or mature miRNA in the cytoplasm through cleavage of different sites in their structures [186]. Recent reports indicate that more than 21 thousand mature miRNA molecules have been isolated in many different species [187].

In cancer, miRNAs are located mostly in cancer-related genomic areas, where miRNA can be either oncogenes or tumor suppressors [188]. Tumor suppressor miRNAs repress the oncogenes that induce tumor proliferation, resistance and metastasis; for example, miRNA-15 and 16 are strong inhibitors of the anti-apoptotic protein Bcl-2 [189]. Many miRNAs are significantly altered in different cancers, including breast [190], colorectal [191], brain, and others [192].

In contrast, the role of miRNA families in EMT development is diverse and controversial. Whereas some miRNAs significantly inhibited adoption of the mesenchymal phenotype in epithelial cells, other miRNAs were involved in the induction and maintenance of EMT [193]. miRNA 200 and miRNA 205 are strong suppressors of EMT and promotors of MET phenotype and produce their effects by directly binding to the promotor region of EMT, inducing transcriptional factors (e.g., snail, ZEB1/2 and twist) [194]. The ectopic overexpression of miRNA200 significantly induced higher E-cadherin levels and significantly reduced the levels of ZEB1/2 [194]. There is a significant reduction in the expression of the miRNA 200 subfamilies in metastatic breast cancer, in tandem with increased expression of the ZEB1/2 [195]. miRNA-30a significantly silenced snail transcriptional factor, inducing significant inhibition of EMT in lung carcinoma [196]. Given that EMT can facilitate cell detachment and motility, and thus cell metastasis, EMT-related miRNAs significantly affect the progression of metastasis [197]. Furthermore, some miRNA subfamilies, such as miRNA-9 [198], miRNA-103/107 [199] and miRNA-155 [200], promote cancer metastasis through the induction of EMT. Indeed, miRNA-9 enhanced the invasive and metastatic properties in breast cancer cell lines by directly binding and inhibiting the transcription of the E-cadherin gene (CDH1) miRNA-103/107 significantly suppressed the inhibition of the inhibitory effect of miRNA-200 by silencing its gene expression [201].

It is possible that the polyphenols produce their beneficial effects by altering the expression levels of several miRNAs [202]. However, only a few studies have investigated the relationship between polyphenols and miRNAs, particularly the reversal of cancer metastasis. One study showed that dietary polyphenols can alter the expression of several miRNAs that control important cellular signaling pathways such as TGF-β and Wnt signaling, which are inducers of EMT [202]. Another study demonstrated that 10 µM of a mixture of isoflavones (70.5% genistein, 26.3% diadzein, 0.3% glycitein) resulted in the significant upregulation of miRNA 200 and miRNA let-7, as well as significant upregulation of E-cadherin and downregulation of mesenchymal markers, including vemintin, ZEB1 and slug in gemcitabine-resistant pancreatic cancer cells [203]. These results tentatively suggest that polyphenols can reverse EMT and cancer metastasis by altering the expression of miRNAs. Therefore, additional preclinical and clinical studies are needed to focus on the role of polyphenols and their analogs in modulating miRNAs expression and its effect on reversing EMT-mediated metastasis. Such studies would enhance our understanding of the detailed mechanisms of action of polyphenols in cancer metastasis.

### 4.2. Whole Extract/Combining Polyphenols Studies

Only a few studies have been focused on elucidating the effects of whole polyphenol extracts on EMT, EMT-related pathways and metastasis, while most investigations determined the efficacy of certain polyphenols isolates or individual compounds. Therefore, future studies should be conducted to determine the efficacy of whole polyphenol extracts in reversing metastasis and identifying the molecular mechanisms. This is important as several studies have reported that the combination of several polyphenolic compounds can further enhance their anticancer efficacy and produce synergistic efficacy against cancer cells proliferation, migration, and invasiveness *in vitro* [204,205]. The combination of polyphenols with other micronutrients, such as vitamin C, was also efficacious [206].

### 4.3. Use of Doses or Concentrations That Resemble Those Consumed from Dietary Sources

Similar to other natural products, polyphenols are present in dietary sources as complexes with other natural products and chemicals. As indicated earlier, polyphenols are typically extensively metabolized, which further limits their bioavailability. The majority of past studies used high concentrations or doses of polyphenols that were significantly higher than what humans consume in their diet or receive in clinical settings. Therefore, future studies should consider the complex nature of polyphenols, as well as their suboptimal pharmacokinetic profile. It is important to use doses or concentrations that more closely resemble those obtained from dietary consumption in both preclinical and clinical studies to further understand potential health benefits of polyphenols in cancer and other diseases.

## 5. Conclusions

In conclusion, the anticancer efficacy of polyphenols may be due in part to their effects on the EMT pathway. Polyphenols may also produce their efficacy by inducing apoptosis, increasing ROS levels and modulating the immune system and other mechanisms as previously discussed in this paper. Notably, EMT is particularly important in mediating cancer cell migration and invasive properties, and this can explain, in part, the antimetastatic efficacy of many polyphenolic compounds and mixtures. Different types and mixtures of polyphenols with versatile structural and chemical properties significantly upregulate epithelial markers such as E-cadherin, while significantly downregulate mesenchymal proteins such as N-cadherin, vimentin, and fibronectin. Polyphenols also significantly alter the expression levels of EMT-inducing transcriptional factors such as snail and twist. Importantly, other signaling pathways that interact with the EMT pathway were also affected, possibly explaining the so-called EMT efficacy of the polyphenols. Although efficacious in limiting the EMT process, dietary polyphenols also have limitations that should be considered, including poor and complex biodistribution, kinetics, and metabolic fate *in vivo*. Polyphenols may have limited oral absorption, undergo extensive metabolic degradation, and significantly interact with other ingested drugs and foods, among others. Many of the studies on the effects of polyphenols on EMT signaling are *in vitro* studies that do not reflect the concentrations achieved *in vivo*. Accordingly, additional *in vivo* studies are needed that focus on using concentrations and doses of polyphenols that reflect those consumed from dietary sources and clinical studies which are safe and equivalent to the achieved *in vivo* levels. The identification of specific cellular targets is another limitation. The specific receptors, proteins or transcriptional factors that are directly affected by polyphenols remain to be elucidated.

## Figures and Tables

**Table 1:** A summary of the effect of selected polyphenolic compounds on the EMT markers and proteins in the related pathways.

Polyphenol subfamily	Member		Effect on EMT markers or related proteins	Studies type
Flavonoids	EGCG		↑ E-cadhrin, ↓ Veminten , Snail	*In vitro*
	Quercetin		↑ E- cadherin ↓ Fibronectin, Vimentin, Twist, Snail, MMP-9, and N-cadherin	*In vitro*
Stilbines	Resveratrol		↑ E-cadherin ↓ N-cadherin and Snail, Vimentin, ZEB 1/2, and TGF-β,	*In vitro*
Phenolic acids	hydroxybenzoic acid	gallic acid	↓ MMP-9, TGF-β, IL-6, β-catenin, NFκB, VEGF	*In vitro*
	hydroxycinnamic acid	caffeic acid	↑ E-cadherin ↓ NF-κB/snail, N-cadherin and Vimentin	*In vitro*
		Ferulic	↑ E-cadherin ↓ P-Smad2/3, Fibronectin, Snail	*In vitro* & *in vivo*
Lignans	Secoisolariciresinol		No data corelate their effect to EMT	
	Matairesinol			

## References

[1] Pandey K.B., Rizvi S.I. (2009). Plant polyphenols as dietary antioxidants in human health and disease. Oxid. Med. Cell. Longev..

[2] Lu J.N., Lee W.S., Kim M.J., Yun J.W., Jung J.H., Yi S.M., Jeong J.-H., Kim H.J., Choi Y.H., Kim G.S. (2014). The inhibitory effect of anthocyanins on Akt on invasion and epithelial-mesenchymal transition is not associated with the anti-EGFR effect of the anthocyanins. Int. J. Oncol..

[3] Kundu J.K., Chun K.-S. (2014). The promise of dried fruits in cancer chemoprevention. Asian Pac. J. Cancer Prev..

[4] Perez-Jimenez J., Neveu V., Vos F., Scalbert A. (2010). Identification of the 100 richest dietary sources of polyphenols: An application of the phenol-explorer database. Eur. J. Clin. Nutr..

[5] Duthie G.G., Duthie S.J., Kyle J.A. (2000). Plant polyphenols in cancer and heart disease: Implications as nutritional antioxidants. Nutr. Res. Rev..

[6] Grosso G., Godos J., Lamuela-Raventos R., Ray S., Micek A., Pajak A., Sciacca S., D’Orazio N., Del Rio D., Galvano F. (2017). A comprehensive meta-analysis on dietary flavonoid and lignan intake and cancer risk: Level of evidence and limitations. Mol. Nutr. Food Res..

[7] Illam S.P., Narayanankutty A., Mathew S.E., Valsalakumari R., Jacob R.M., Raghavamenon A.C. (2017). Epithelial Mesenchymal Transition in Cancer Progression: Preventive Phytochemicals. Recent Pat. Anti-Cancer Drug Discov..

[8] Kim E.K., Choi E.J., Debnath T. (2016). Role of phytochemicals in the inhibition of epithelial-mesenchymal transition in cancer metastasis. Food Funct..

[9] Kalluri R., Weinberg R.A. (2009). The basics of epithelial-mesenchymal transition. J. Clin. Investig..

[10] Thiery J.P. (2002). Epithelial–mesenchymal transitions in tumour progression. Nat. Rev. Cancer.

[11] Manach C., Williamson G., Morand C., Scalbert A., Rémésy C. (2005). Bioavailability and bioefficacy of polyphenols in humans. I. Review of 97 bioavailability studies. Am. J. Clin. Nutr..

[12] Scalbert A., Johnson I.T., Saltmarsh M. (2005). Polyphenols: Antioxidants and beyond. Am. J. Clin. Nutr..

[13] Manach C., Scalbert A., Morand C., Rémésy C., Jiménez L. (2004). Polyphenols: Food sources and bioavailability. Am. J. Clin. Nutr..

[14] Kahkonen M.P., Hopia A.I., Vuorela H.J., Rauha J.P., Pihlaja K., Kujala T.S., Heinonen M. (1999). Antioxidant activity of plant extracts containing phenolic compounds. J. Agric. Food Chem..

[15] Harborne J.B., Williams C.A. (2000). Advances in flavonoid research since 1992. Phytochemistry.

[16] El Gharras H. (2009). Polyphenols: Food sources, properties and applications—A review. Int. J. Food Sci. Technol..

[17] Tsao R. (2010). Chemistry and biochemistry of dietary polyphenols. Nutrients.

[18] Barbieri R., Coppo E., Marchese A., Daglia M., Sobarzo-Sánchez E., Nabavi S.F., Nabavi S.M. (2017). Phytochemicals for human disease: An update on plant-derived compounds antibacterial activity. Microbiol. Res..

[19] Skalicka-Wozniak K., Orhan I.E., Cordell G.A., Nabavi S.M., Budzynska B. (2016). Implication of coumarins towards central nervous system disorders. Pharmacol. Res..

[20] Menezes R., Rodriguez-Mateos A., Kaltsatou A., González-Sarrías A., Greyling A., Giannaki C., Andres-Lacueva C., Milenkovic D., Gibney E.R., Dumont J. (2017). Impact of flavonols on cardiometabolic biomarkers: A meta-analysis of randomized controlled human trials to explore the role of inter-individual variability. Nutrients.

[21] Hostetler G.L., Ralston R.A., Schwartz S.J. (2017). Flavones: Food sources, bioavailability, metabolism, and bioactivity. Advances in nutrition (Bethesda, Md.).

[22] Vandeputte O.M., Kiendrebeogo M., Rasamiravaka T., Stevigny C., Duez P., Rajaonson S., Diallo B., Mol A., Baucher M., El Jaziri M. (2011). The flavanone naringenin reduces the production of quorum sensing-controlled virulence factors in pseudomonas aeruginosa Pao1. Microbiology.

[23] Adlercreutz H., Mazur W. (1997). Phyto-oestrogens and western diseases. Ann. Med..

[24] Ferrazzano G.F., Amato I., Ingenito A., Zarrelli A., Pinto G., Pollio A. (2011). Plant polyphenols and their anti-cariogenic properties: A review. Molecules.

[25] Hara Y., Luo S., Wickremasinghe R., Yamanishi T. (1995). Special issue on tea. Food Rev. Int..

[26] Prior R.L., Wu X. (2006). Anthocyanins: Structural characteristics that result in unique metabolic patterns and biological activities. Free Radic. Res..

[27] Wang Y., Ho C.T. (2009). Polyphenolic chemistry of tea and coffee: A century of progress. J. Agric. Food Chem..

[28] Clifford M.N. (1999). Chlorogenic acids and other cinnamates—Nature, occurrence and dietary burden. J. Sci. Food Agric..

[29] Jang M., Cai L., Udeani G.O., Slowing K.V., Thomas C.F., Beecher C.W.W., Fong H.H.S., Farnsworth N.R., Kinghorn A.D., Mehta R.G. (1997). Cancer chemopreventive activity of resveratrol, a natural product derived from grapes. Science.

[30] Scalbert A., Williamson G. (2000). Dietary intake and bioavailability of polyphenols. J. Nutr..

[31] Sies H., Schewe T., Heiss C., Kelm M. (2005). Cocoa polyphenols and inflammatory mediators. Am. J. Clin. Nutr..

[32] Vita J.A. (2005). Polyphenols and cardiovascular disease: Effects on endothelial and platelet function. Am. J. Clin. Nutr..

[33] Cho Y., Schiller N., Kahng H., Oh K. (2007). Cellular responses and proteomic analysis of *Escherichia coli* exposed to green tea polyphenols. Curr. Microbiol..

[34] Hubert P.A., Lee S.G., Lee S.-K., Chun O.K. (2014). Dietary polyphenols, berries, and age-related bone loss: A review based on human, animal, and cell studies. Antioxidants.

[35] Lambert J.D., Hong J., Yang G.Y., Liao J., Yang C.S. (2005). Inhibition of carcinogenesis by polyphenols: Evidence from laboratory investigations. Am. J. Clin. Nutr..

[36] Who J., Consultation F.E. (2003). Diet, nutrition and the prevention of chronic diseases. World Health Organ. Tech. Rep. Ser..

[37] Rice-Evans C. (2001). Flavonoid antioxidants. Curr. Med. Chem..

[38] Upadhyay S., Dixit M. (2015). Role of polyphenols and other phytochemicals on molecular signaling. Oxid. Med. Cell. Longev..

[39] Rechner A.R., Kuhnle G., Bremner P., Hubbard G.P., Moore K.P., Rice-Evans C.A. (2002). The metabolic fate of dietary polyphenols in humans. Free Radic. Biol. Med..

[40] Manach C., Morand C., Crespy V., Demigné C., Texier O., Régérat F., Rémésy C. (1998). Quercetin is recovered in human plasma as conjugated derivatives which retain antioxidant properties. FEBS Lett..

[41] Farber E. (1984). The multistep nature of cancer development. Cancer Res..

[42] Ramos S. (2008). Cancer chemoprevention and chemotherapy: Dietary polyphenols and signaling pathways. Mol. Nutr. Food Res..

[43] Fresco P., Borges F., Marques M.P., Diniz C. (2010). The anticancer properties of dietary polyphenols and its relation with apoptosis. Curr. Pharm. Des..

[44] Amararathna M., Johnston M.R., Rupasinghe H.P. (2016). Plant polyphenols as chemopreventive agents for lung cancer. Int. J. Mol. Sci..

[45] American Cancer Society (2017). Cancer Facts & Figures 2017. Nutrition and Physical Activities.

[46] Turrini E., Ferruzzi L., Fimognari C. (2015). Potential effects of pomegranate polyphenols in cancer prevention and therapy. Oxid. Med. Cell. Longev..

[47] Wenzel U., Kuntz S., Brendel M.D., Daniel H. (2000). Dietary flavone is a potent apoptosis inducer in human colon carcinoma cells. Cancer Res..

[48] Yang C.S., Landau J.M., Huang M.T., Newmark H.L. (2001). Inhibition of carcinogenesis by dietary polyphenolic compounds. Ann. Rev. Nutr..

[49] Hadi S., Asad S., Singh S., Ahmad A. (2000). Putative mechanism for anticancer and apoptosis-inducing properties of plant-derived polyphenolic compounds. IUBMB Life Sci..

[50] Wessner B., Strasser E.M., Koitz N., Schmuckenschlager C., Unger-Manhart N., Roth E. (2007). Green tea polyphenol administration partly ameliorates chemotherapy-induced side effects in the small intestine of mice. J. Nutr..

[51] Harper C.E., Patel B.B., Wang J., Eltoum I.A., Lamartiniere C.A. (2007). Epigallocatechin-3-gallate suppresses early stage, but not late stage prostate cancer in tramp mice: Mechanisms of action. Prostate.

[52] Chuang S.E., Cheng A.L., Lin J.K., Kuo M.L. (2000). Inhibition by curcumin of diethylnitrosamine-induced hepatic hyperplasia, inflammation, cellular gene products and cell-cycle-related proteins in rats. Food Chem. Toxicol..

[53] Surh Y.J. (2003). Cancer chemoprevention with dietary phytochemicals. Nat. Rev. Cancer.

[54] Weng C.-J., Yen G.-C. (2012). Chemopreventive effects of dietary phytochemicals against cancer invasion and metastasis: Phenolic acids, monophenol, polyphenol, and their derivatives. Cancer Treat. Rev..

[55] Chen D., Daniel K.G., Kuhn D.J., Kazi A., Bhuiyan M., Li L., Wang Z., Wan S.B., Lam W.H., Chan T.H. (2004). Green tea and tea polyphenols in cancer prevention. Front Biosci..

[56] Link A., Balaguer F., Goel A. (2010). Cancer chemoprevention by dietary polyphenols: Promising role for epigenetics. Biochem. Pharmacol..

[57] Tsuji P.A., Walle T. (2006). Inhibition of benzo [a] pyrene-activating enzymes and DNA binding in human bronchial epithelial BEAS-2B cells by methoxylated flavonoids. Carcinogenesis.

[58] Zhai X., Lin M., Zhang F., Hu Y., Xu X., Li Y., Liu K., Ma X., Tian X., Yao J. (2013). Dietary flavonoid genistein induces NRF2 and Phase II detoxification gene expression via ERKS and PKC pathways and protects against oxidative stress in CACO-2 cells. Mol. Nutr. Food Res..

[59] Murakami C., Hirakawa Y., Nakano Y., Yoshida H. (2002). Effects of epigallocatechin 3-O-gallate on cellular antioxidative system in HepG2 cells. J. Nutr. Sci. Vitaminol..

[60] Schwarz D., Roots I. (2003). In vitro assessment of inhibition by natural polyphenols of metabolic activation of procarcinogens by human CYP1A1. Biochem. Biophys. Res. Commun..

[61] Howells L.M., Mitra A., Manson M.M. (2007). Comparison of oxaliplatin-and curcumin-mediated antiproliferative effects in colorectal cell lines. Int. J. Cancer.

[62] Nakazato T., Ito K., Ikeda Y., Kizaki M. (2005). Green tea component, catechin, induces apoptosis of human malignant B cells via production of reactive oxygen species. Clin. Cancer Res..

[63] Balasubramanian S., Efimova T., Eckert R.L. (2002). Green tea polyphenol stimulates a Ras, Mekk1, Mek3, and p38 cascade to increase activator protein 1 factor-dependent involucrin gene expression in normal human keratinocytes. J. Biol. Chem..

[64] Gong L., Li Y., Nedeljkovic-Kurepa A., Sarkar F.H. (2003). Inactivation of NF-κB by genistein is mediated via Akt signaling pathway in breast cancer cells. Oncogene.

[65] Ye F., Wu J., Dunn T., Yi J., Tong X., Zhang D. (2004). Inhibition of cyclooxygenase-2 activity in head and neck cancer cells by genistein. Cancer Lett..

[66] Adams L.S., Seeram N.P., Aggarwal B.B., Takada Y., Sand D., Heber D. (2006). Pomegranate juice, total pomegranate ellagitannins, and punicalagin suppress inflammatory cell signaling in colon cancer cells. J. Agric. Food Chem..

[67] Kao Y.-L., Kuo Y.-M., Lee Y.-R., Yang S.-F., Chen W.-R., Lee H.-J. (2015). Apple polyphenol induces cell apoptosis, cell cycle arrest at G2/m phase, and mitotic catastrophe in human bladder transitional carcinoma cells. J. Funct. Foods.

[68] Singh M., Singh R., Bhui K., Tyagi S., Mahmood Z., Shukla Y. (2011). Tea polyphenols induce apoptosis through mitochondrial pathway and by inhibiting nuclear factor-kappa B and Akt activation in human cervical cancer cells. Oncol. Res..

[69] Monasterio A., Urdaci M.C., Pinchuk I.V., Lopez-Moratalla N., Martinez-Irujo J.J. (2004). Flavonoids induce apoptosis in human leukemia u937 cells through caspase-and caspase-calpain-dependent pathways. Nutr. Cancer.

[70] Brusselmans K., Vrolix R., Verhoeven G., Swinnen J.V. (2005). Induction of cancer cell apoptosis by flavonoids is associated with their ability to inhibit fatty acid synthase activity. J. Biol. Chem..

[71] Lee S.H., Yumnam S., Hong G.E., Raha S., Saralamma V.V., Lee H.J., Heo J.D., Lee S.J., Lee W.S., Kim E.H. (2015). Flavonoids of korean citrus *aurantium* L. induce apoptosis via intrinsic pathway in human hepatoblastoma HepG2 cells. Phytother. Res. PTR.

[72] Miquel J., Bernd A., Sempere J.M., Diaz-Alperi J., Ramirez A. (2002). The curcuma antioxidants: Pharmacological effects and prospects for future clinical use. A review. Arch. Gerontol. Geriatr..

[73] Sarkar F.H., Li Y., Wang Z., Kong D. (2010). The role of nutraceuticals in the regulation of Wnt and hedgehog signaling in cancer. Cancer Metastasis Rev..

[74] George V.C., Dellaire G., Rupasinghe H.P.V. (2017). Plant flavonoids in cancer chemoprevention: Role in genome stability. J. Nutr. Biochem..

[75] Garcia R., Gonzalez C.A., Agudo A., Riboli E. (1999). High intake of specific carotenoids and flavonoids does not reduce the risk of bladder cancer. Nutr. Cancer.

[76] Arts I.C., Hollman P.C., Bueno De Mesquita H.B., Feskens E.J., Kromhout D. (2001). Dietary catechins and epithelial cancer incidence: The zutphen elderly study. Int. J. Cancer.

[77] Mehlen P., Puisieux A. (2006). Metastasis: A question of life or death. Nature Reviews Cancer.

[78] Kohn E.C. (1992). Development and prevention of metastasis. Anticancer Res..

[79] Ali S., Lazennec G. (2007). Chemokines: Novel targets for breast cancer metastasis. Cancer Metastasis Rev..

[80] Nguyen D.X., Bos P.D., Massague J. (2009). Metastasis: From dissemination to organ-specific colonization. Nat. Rev. Cancer.

[81] Jiang W.G., Sanders A.J., Katoh M., Ungefroren H., Gieseler F., Prince M., Thompson S.K., Zollo M., Spano D., Dhawan P. (2015). Tissue invasion and metastasis: Molecular, biological and clinical perspectives. Semin. Cancer Biol..

[82] Shih J.-Y., Yang S.-C., Hong T.-M., Yuan A., Chen J.J., Yu C.-J., Chang Y.-L., Lee Y.-C., Peck K., Wu C.-W. (2001). Collapsin response mediator protein-1 and the invasion and metastasis of cancer cells. J. Natl. Cancer Inst..

[83] Orr F.W., Wang H.H., Lafrenie R.M., Scherbarth S., Nance D.M. (2000). Interactions between cancer cells and the endothelium in metastasis. J. Pathol..

[84] Clark E.A., Golub T.R., Lander E.S., Hynes R.O. (2000). Genomic analysis of metastasis reveals an essential role for RhoC. Nature.

[85] Mikami S., Mizuno R., Kosaka T., Saya H., Oya M., Okada Y. (2015). Expression of TNF-α and CD44 is implicated in poor prognosis, cancer cell invasion, metastasis and resistance to the sunitinib treatment in clear cell renal cell carcinomas. Int. J. Cancer.

[86] Deryugina E.I., Quigley J.P. (2015). Tumor angiogenesis: Mmp-mediated induction of intravasation- and metastasis-sustaining neovasculature. Matrix Biol..

[87] Slaney C.Y., Rautela J., Parker B.S. (2013). The emerging role of immunosurveillance in dictating metastatic spread in breast cancer. Cancer Res..

[88] Gordon M.A., Zhang W., Yang D., Iqbal S., El-Khouiery A., Nagashima F., Lurje G., Labonte M., Wilson P., Sherrod A. (2011). Gender-specific genomic profiling in metastatic colorectal cancer patients treated with 5-fluorouracil and oxaliplatin. Pharmacogenomics.

[89] Nguyen D.X., Massague J. (2007). Genetic determinants of cancer metastasis. Nat. Rev. Genet..

[90] National Cancer Institute (2017). Metastatic Cancer.

[91] Liang X. (2011). EMT: New signals from the invasive front. Oral Oncol..

[92] Polyak K., Weinberg R.A. (2009). Transitions between epithelial and mesenchymal states: Acquisition of malignant and stem cell traits. Nat. Rev. Cancer.

[93] Lamouille S., Xu J., Derynck R. (2014). Molecular mechanisms of epithelial—Mesenchymal transition. Nat. Rev. Mol. Cell Biol..

[94] Onder T.T., Gupta P.B., Mani S.A., Yang J., Lander E.S., Weinberg R.A. (2008). Loss of E-cadherin promotes metastasis via multiple downstream transcriptional pathways. Cancer Res..

[95] Nelson W.J., Nusse R. (2004). Convergence of Wnt, ß-catenin, and cadherin pathways. Science.

[96] Xie L., Law B.K., Chytil A.M., Brown K.A., Aakre M.E., Moses H.L. (2004). Activation of the Erk pathway is required for TGF-β1-induced EMT in vitro. Neoplasia.

[97] Boyer B., Vallés A.M., Edme N. (2000). Induction and regulation of epithelial—Mesenchymal transitions. Biochem. Pharmacol..

[98] Willis B.C., Borok Z. (2007). TGF-β-induced EMT: Mechanisms and implications for fibrotic lung disease. Am. J. Physiol.-Lung Cell. Mol. Physiol..

[99] Graham T.R., Zhau H.E., Odero-Marah V.A., Osunkoya A.O., Kimbro K.S., Tighiouart M., Liu T., Simons J.W., O’Regan R.M. (2008). Insulin-like growth factor-I–dependent up-regulation of ZEB1 drives epithelial-to-mesenchymal transition in human prostate cancer cells. Cancer Res..

[100] Lo H.-W., Hsu S.-C., Xia W., Cao X., Shih J.-Y., Wei Y., Abbruzzese J.L., Hortobagyi G.N., Hung M.-C. (2007). Epidermal growth factor receptor cooperates with signal transducer and activator of transcription 3 to induce epithelial-mesenchymal transition in cancer cells via up-regulation of Twist gene expression. Cancer Res..

[101] Kong D., Wang Z., Sarkar S.H., Li Y., Banerjee S., Saliganan A., Kim H.R.C., Cher M.L., Sarkar F.H. (2008). Platelet-derived growth factor-d overexpression contributes to epithelial-mesenchymal transition of PC3 prostate cancer cells. Stem Cells.

[102] Julien S., Puig I., Caretti E., Bonaventure J., Nelles L., Van Roy F., Dargemont C., De Herreros A.G., Bellacosa A., Larue L. (2007). Activation of NF-κb by Akt upregulates snail expression and induces epithelium mesenchyme transition. Oncogene.

[103] Grille S.J., Bellacosa A., Upson J., Klein-Szanto A.J., Van Roy F., Lee-Kwon W., Donowitz M., Tsichlis P.N., Larue L. (2003). The protein kinase Akt induces epithelial mesenchymal transition and promotes enhanced motility and invasiveness of squamous cell carcinoma lines. Cancer Res..

[104] Kim K., Lu Z., Hay E.D. (2002). Direct evidence for a role of β-catenin/lef-1 signaling pathway in induction of EMT. Cell Biol. Int..

[105] Hu C.-T., Wu J.-R., Chang T.Y., Cheng C.-C., Wu W.-S. (2008). The transcriptional factor snail simultaneously triggers cell cycle arrest and migration of human hepatoma HepG2. J. Biomed. Sci..

[106] Peinado H., Marin F., Cubillo E., Stark H.-J., Fusenig N., Nieto M.A., Cano A. (2004). Snail and E47 repressors of E-cadherin induce distinct invasive and angiogenic properties in vivo. J. Cell Sci..

[107] Spaderna S., Schmalhofer O., Wahlbuhl M., Dimmler A., Bauer K., Sultan A., Hlubek F., Jung A., Strand D., Eger A. (2008). The transcriptional repressor Zeb1 promotes metastasis and loss of cell polarity in cancer. Cancer Res..

[108] Yang J., Mani S.A., Donaher J.L., Ramaswamy S., Itzykson R.A., Come C., Savagner P., Gitelman I., Richardson A., Weinberg R.A. (2004). Twist, a master regulator of morphogenesis, plays an essential role in tumor metastasis. Cell.

[109] Yao D., Dai C., Peng S. (2011). Mechanism of the mesenchymal–epithelial transition and its relationship with metastatic tumor formation. Mol. Cancer Res..

[110] Brembeck F.H., Rosário M., Birchmeier W. (2006). Balancing cell adhesion and wnt signaling, the key role of β-catenin. Curr. Opin. Genet. Dev..

[111] MacDonald B.T., Tamai K., He X. (2009). Wnt/β-catenin signaling: Components, mechanisms, and diseases. Dev. Cell.

[112] Clevers H. (2006). Wnt/β-catenin signaling in development and disease. Cell.

[113] Eastman Q., Grosschedl R. (1999). Regulation of LEF-1/TCF transcription factors by Wnt and other signals. Curr. Opin. Cell Biol..

[114] Roose J., Huls G., Van Beest M., Moerer P., Van Der Horn K., Goldschmeding R., Logtenberg T., Clevers H. (1999). Synergy between tumor suppressor APC and the β-catenin-TCF4 target TCF1. Science.

[115] Van De Wetering M., Sancho E., Verweij C., De Lau W., Oving I., Hurlstone A., Van Der Horn K., Batlle E., Coudreuse D., Haramis A.-P. (2002). The β-catenin/TCF-4 complex imposes a crypt progenitor phenotype on colorectal cancer cells. Cell.

[116] Davidson G., Wu W., Shen J., Bilic J., Fenger U., Stannek P., Glinka A., Niehrs C. (2005). Casein kinase 1 γ couples Wnt receptor activation to cytoplasmic signal transduction. Nature.

[117] Orsulic S., Huber O., Aberle H., Arnold S., Kemler R. (1999). E-cadherin binding prevents beta-catenin nuclear localization and beta-catenin/LEF-1-mediated transactivation. J. Cell Sci..

[118] Chang Y.-W., Su Y.-J., Hsiao M., Wei K.-C., Lin W.-H., Liang C.-J., Chen S.-C., Lee J.-L. (2015). Diverse targets of β-catenin during the epithelial–mesenchymal transition define cancer stem cells and predict disease relapse. Cancer Res..

[119] Ozawa M., Baribault H., Kemler R. (1989). The cytoplasmic domain of the cell adhesion molecule uvomorulin associates with three independent proteins structurally related in different species. EMBO J..

[120] Cano A., Pérez-Moreno M.A., Rodrigo I., Locascio A., Blanco M.J., del Barrio M.G., Portillo F., Nieto M.A. (2000). The transcription factor snail controls epithelial—Mesenchymal transitions by repressing E-cadherin expression. Nat. Cell Biol..

[121] Stemmer V., De Craene B., Berx G., Behrens J. (2008). Snail promotes Wnt target gene expression and interacts with β-catenin. Oncogene.

[122] Yook J.I., Li X.-Y., Ota I., Fearon E.R., Weiss S.J. (2005). Wnt-dependent regulation of the E-cadherin repressor snail. J. Biol. Chem..

[123] Zhou B.P., Deng J., Xia W., Xu J., Li Y.M., Gunduz M., Hung M.-C. (2004). Dual regulation of snail by GSK-3β-mediated phosphorylation in control of epithelial–mesenchymal transition. Nat. Cell Biol..

[124] Zucchini-Pascal N., Peyre L., Rahmani R. (2013). Crosstalk between beta-catenin and snail in the induction of epithelial to mesenchymal transition in hepatocarcinoma: Role of the Erk1/2 pathway. Int. J. Mol. Sci..

[125] Huang J., Li H., Ren G. (2015). Epithelial-mesenchymal transition and drug resistance in breast cancer (review). Int. J. Oncol..

[126] Sommers C.L., Heckford S.E., Skerker J.M., Worland P., Torri J.A., Thompson E.W., Byers S.W., Gelmann E.P. (1992). Loss of epithelial markers and acquisition of vimentin expression in adriamycin-and vinblastine-resistant human breast cancer cell lines. Cancer Res..

[127] Fischer K.R., Durrans A., Lee S., Sheng J., Li F., Wong S.T., Choi H., El Rayes T., Ryu S., Troeger J. (2015). Epithelial-to-mesenchymal transition is not required for lung metastasis but contributes to chemoresistance. Nature.

[128] Du B., Shim J.S. (2016). Targeting epithelial–mesenchymal transition (EMT) to overcome drug resistance in cancer. Molecules.

[129] Kampa M., Nifli A.P., Notas G., Castanas E. (2007). Polyphenols and cancer cell growth. Rev. Physiol. Biochem. Pharmacol..

[130] Zhou Q., Bennett L.L., Zhou S. (2016). Multifaceted ability of naturally occurring polyphenols against metastatic cancer. Clin. Exp. Pharmacol. Physiol..

[131] Kita Y., Miura Y., Yagasaki K. (2012). Antiproliferative and anti-invasive effect of piceatannol, a polyphenol present in grapes and wine, against hepatoma AH109A cells. BioMed Res. Int..

[132] Lee S.H., Jaganath I.B., Wang S.M., Sekaran S.D. (2011). Antimetastatic effects of phyllanthus on human lung (A549) and breast (MCF-7) cancer cell lines. PLoS ONE.

[133] Vergara D., Simeone P., Bettini S., Tinelli A., Valli L., Storelli C., Leo S., Santino A., Maffia M. (2014). Antitumor activity of the dietary diterpene carnosol against a panel of human cancer cell lines. Food Funct..

[134] Lee S.J., Chung I.M., Kim M.Y., Park K.D., Park W.W., Moon H.I. (2009). Inhibition of lung metastasis in mice by oligonol. Phytother. Res..

[135] Ho Y.C., Yang S.F., Peng C.Y., Chou M.Y., Chang Y.C. (2007). Epigallocatechin-3-gallate inhibits the invasion of human oral cancer cells and decreases the productions of matrix metalloproteinases and urokinase-plasminogen activator. J. Oral Pathol. Med..

[136] Sun C.-Y., Hu Y., Guo T., Wang H.-F., Zhang X.-P., He W.-J., Tan H. (2006). Resveratrol as a novel agent for treatment of multiple myeloma with matrix metalloproteinase inhibitory activity. Acta Pharmacol. Sin..

[137] Bigelow R.L.H., Cardelli J.A. (2006). The green tea catechins, (−)-epigallocatechin-3-gallate (EGCG) and (−)-epicatechin-3-gallate (ECG), inhibit HGF//Met signaling in immortalized and tumorigenic breast epithelial cells. Oncogene.

[138] Milligan S.A., Burke P., Coleman D.T., Bigelow R.L., Steffan J.J., Carroll J.L., Williams B.J., Cardelli J.A. (2009). The green tea polyphenol EGCG potentiates the antiproliferative activity of c-met and epidermal growth factor receptor inhibitors in non–small cell lung cancer cells. Clin. Cancer Res..

[139] Bachmeier B.E., Nerlich A.G., Iancu C.M., Cilli M., Schleicher E., Vené R., Dell’Eva R., Jochum M., Albini A., Pfeffer U. (2007). The chemopreventive polyphenol curcumin prevents hematogenous breast cancer metastases in immunodeficient mice. Cell. Physiol. Biochem..

[140] Sung B., Pandey M.K., Nakajima Y., Nishida H., Konishi T., Chaturvedi M.M., Aggarwal B.B. (2008). Identification of a novel blocker of Iκbα kinase activation that enhances apoptosis and inhibits proliferation and invasion by suppressing nuclear factor-κb. Mol. Cancer Ther..

[141] Zetter P., Bruce R. (1998). Angiogenesis and tumor metastasis. Ann. Rev. Med..

[142] Terzuoli E., Donnini S., Giachetti A., Iñiguez M.A., Fresno M., Melillo G., Ziche M. (2010). Inhibition of hypoxia inducible factor-1α by dihydroxyphenylethanol, a product from olive oil, blocks microsomal prostaglandin-E synthase-1/vascular endothelial growth factor expression and reduces tumor angiogenesis. Clin. Cancer Res..

[143] Ko Y.S., Lee W.S., Panchanathan R., Joo Y.N., Choi Y.H., Kim G.S., Jung J.M., Ryu C.H., Shin S.C., Kim H.J. (2016). Polyphenols from *Artemisia annua* L. inhibit adhesion and EMT of highly metastatic breast cancer cells MDA-MB-231. Phytother. Res..

[144] Ko Y.S., Lee W.S., Joo Y.N., Choi Y.H., Kim G.S., Jung J.-M., Ryu C.H., Shin S.C., Kim H.J. (2015). Polyphenol mixtures of euphorbia supina the inhibit invasion and metastasis of highly metastatic breast cancer MDA-MB-231 cells. Oncol. Rep..

[145] Zhang Z., Chen H., Xu C., Song L., Huang L., Lai Y., Wang Y., Chen H., Gu D., Ren L. (2016). Curcumin inhibits tumor epithelial-mesenchymal transition by downregulating the Wnt signaling pathway and upregulating NKD2 expression in colon cancer cells. Oncol. Rep..

[146] Amawi H., Ashby C.R., Tiwari A.K. (2017). Cancer chemoprevention through dietary flavonoids: What’s limiting?. Chin. J. Cancer.

[147] Hertog M.G., Kromhout D., Aravanis C., Blackburn H., Buzina R., Fidanza F., Giampaoli S., Jansen A., Menotti A., Nedeljkovic S. (1995). Flavonoid intake and long-term risk of coronary heart disease and cancer in the seven countries study. Arch. Int. Med..

[148] Nijveldt R.J., Van Nood E., Van Hoorn D.E., Boelens P.G., Van Norren K., Van Leeuwen P.A. (2001). Flavonoids: A review of probable mechanisms of action and potential applications. Am. J. Clin. Nutr..

[149] Kang J., Kim E., Kim W., Seong K.M., Youn H., Kim J.W., Kim J., Youn B. (2013). Rhamnetin and cirsiliol induce radiosensitization and inhibition of epithelial-mesenchymal transition (EMT) by MIR-34A-mediated suppression of notch-1 expression in non-small cell lung cancer cell lines. J. Biol. Chem..

[150] Lin C.-H., Shen Y.-A., Hung P.-H., Yu Y.-B., Chen Y.-J. (2012). Epigallocathechin gallate, polyphenol present in green tea, inhibits stem-like characteristics and epithelial-mesenchymal transition in nasopharyngeal cancer cell lines. BMC Complement. Altern. Med..

[151] Lin Y.S., Tsai P.H., Kandaswami C.C., Cheng C.H., Ke F.C., Lee P.P., Hwang J.J., Lee M.T. (2011). Effects of dietary flavonoids, luteolin, and quercetin on the reversal of epithelial-mesenchymal transition in A431 epidermal cancer cells. Cancer Sci..

[152] Chong J., Poutaraud A., Hugueney P. (2009). Metabolism and roles of stilbenes in plants. Plant Sci..

[153] Ji Q., Liu X., Han Z., Zhou L., Sui H., Yan L., Jiang H., Ren J., Cai J., Li Q. (2015). Resveratrol suppresses epithelial-to-mesenchymal transition in colorectal cancer through TGF-β1/Smads signaling pathway mediated snail/E-cadherin expression. BMC Cancer.

[154] Vergara D., Valente C.M., Tinelli A., Siciliano C., Lorusso V., Acierno R., Giovinazzo G., Santino A., Storelli C., Maffia M. (2011). Resveratrol inhibits the epidermal growth factor-induced epithelial mesenchymal transition in MCF-7 cells. Cancer Lett..

[155] Gao Q., Yuan Y., Gan H.Z., Peng Q. (2015). Resveratrol inhibits the hedgehog signaling pathway and epithelial-mesenchymal transition and suppresses gastric cancer invasion and metastasis. Oncol. Lett..

[156] Baribeau S., Chaudhry P., Parent S., Asselin É. (2014). Resveratrol inhibits cisplatin-induced epithelial-to-mesenchymal transition in ovarian cancer cell lines. PLoS ONE.

[157] Vergara D., De Domenico S., Tinelli A., Stanca A., Del Mercato L.L., Giudetti A.M., Simeone P., Guazzelli N., Lessi M., Manzini C. (2017). Anticancer effects of novel resveratrol analogues on human ovarian cancer cells. Mol. Biosyst..

[158] Zhou Y., Zheng J., Li Y., Xu D.-P., Li S., Chen Y.-M., Li H.-B. (2016). Natural polyphenols for prevention and treatment of cancer. Nutrients.

[159] Lall R.K., Syed D.N., Adhami V.M., Khan M.I., Mukhtar H. (2015). Dietary polyphenols in prevention and treatment of prostate cancer. Int. J. Mol. Sci..

[160] Carocho M., Ferreira I.C. (2013). The role of phenolic compounds in the fight against cancer—A review. Anti-Cancer Agents Med. Chem..

[161] Anantharaju P.G., Gowda P.C., Vimalambike M.G., Madhunapantula S.V. (2016). An overview on the role of dietary phenolics for the treatment of cancers. Nutr. J..

[162] Niero E.L., Machado-Santelli G.M. (2013). Cinnamic acid induces apoptotic cell death and cytoskeleton disruption in human melanoma cells. J. Exp. Clin. Cancer Res..

[163] Zhao B., Hu M. (2013). Gallic acid reduces cell viability, proliferation, invasion and angiogenesis in human cervical cancer cells. Oncol. Lett..

[164] Rajendra Prasad N., Karthikeyan A., Karthikeyan S., Reddy B.V. (2011). Inhibitory effect of caffeic acid on cancer cell proliferation by oxidative mechanism in human HT-1080 fibrosarcoma cell line. Mol. Cell. Biochem..

[165] Zhang X., Lin D., Jiang R., Li H., Wan J., Li H. (2016). Ferulic acid exerts antitumor activity and inhibits metastasis in breast cancer cells by regulating epithelial to mesenchymal transition. Oncol. Rep..

[166] Wu Y., He L., Zhang L., Chen J., Yi Z., Zhang J., Liu M., Pang X. (2011). Anacardic acid (6-pentadecylsalicylic acid) inhibits tumor angiogenesis by targeting Src/Fak/Rho GTPases signaling pathway. J. Pharmacol. Exp. Ther..

[167] Yao K., Jiang X., He L., Tang Y., Yin G., Zeng Q., Jiang Z., Tan J. (2015). Anacardic acid sensitizes prostate cancer cells to radiation therapy by regulating h2ax expression. Int. J. Clin. Exp. Pathol..

[168] Yang Y., Li Y., Wang K., Wang Y., Yin W., Li L. (2013). P38/NF-kb-/Snail pathway is involved in caffeic acid-induced inhibition of cancer stem cells-like properties and migratory capacity in malignant human keratinocyte. PLoS ONE.

[169] Wei M.G., Sun W., He W.M., Ni L., Yang Y.Y. (2015). Ferulic acid attenuates TGF-beta1-induced renal cellular fibrosis in NRK-52E cells by inhibiting smad/ILK/snail pathway. Evid.-Based Complement. Altern. Med..

[170] Faried A., Kurnia D., Faried L.S., Usman N., Miyazaki T., Kato H., Kuwano H. (2007). Anticancer effects of gallic acid isolated from indonesian herbal medicine, *Phaleria macrocarpa* (Scheff.) Boerl, on human cancer cell lines. Int. J. Oncol..

[171] Su T.R., Lin J.J., Tsai C.C., Huang T.K., Yang Z.Y., Wu M.O., Zheng Y.Q., Su C.C., Wu Y.J. (2013). Inhibition of melanogenesis by gallic acid: Possible involvement of the pi3k/akt, mek/erk and wnt/beta-catenin signaling pathways in b16f10 cells. Int. J. of Mol. Sci..

[172] Wang L., Li W., Lin M., Garcia M., Mulholland D., Lilly M., Martins-Green M. (2014). Luteolin, ellagic acid and punicic acid are natural products that inhibit prostate cancer metastasis. Carcinogenesis.

[173] Lo C., Lai T.Y., Yang J.S., Yang J.H., Ma Y.S., Weng S.W., Lin H.Y., Chen H.Y., Lin J.G., Chung J.G. (2011). Gallic acid inhibits the migration and invasion of A375.S2 human melanoma cells through the inhibition of matrix metalloproteinase-2 and Ras. Melanoma Res..

[174] Teponno R.B., Kusari S., Spiteller M. (2016). Recent advances in research on lignans and neolignans. Nat. prod. Rep..

[175] Milder I.E., Arts I.C., van de Putte B., Venema D.P., Hollman P.C. (2005). Lignan contents of Dutch plant foods: A database including lariciresinol, pinoresinol, secoisolariciresinol and matairesinol. Br. J. Nutr..

[176] Demark-Wahnefried W., Polascik T.J., George S.L., Switzer B.R., Madden J.F., Ruffin M.T.T., Snyder D.C., Owzar K., Hars V., Albala D.M. (2008). Flaxseed supplementation (not dietary fat restriction) reduces prostate cancer proliferation rates in men presurgery. Cancer Epidemiol. Prev. Biomark..

[177] Alphonse P., Aluko R. (2015). A review on the anti-carcinogenic and anti-metastatic effects of flax seed lignan secolariciresinol diglucoside (SDG). Discov. Phytomed..

[178] Li D., Yee J.A., Thompson L.U., Yan L. (1999). Dietary supplementation with secoisolariciresinol diglycoside (SDG) reduces experimental metastasis of melanoma cells in mice. Cancer Lett..

[179] Wang L., Chen J., Thompson L.U. (2005). The inhibitory effect of flaxseed on the growth and metastasisof estrogen receptor negative human breast cancer xenograftsis attributed to both its lignan and oil components. Int. J. Cancer.

[180] Pandima Devi K., Rajavel T., Daglia M., Nabavi S.F., Bishayee A., Nabavi S.M. (2017). Targeting mirnas by polyphenols: Novel therapeutic strategy for cancer. Semin. Cancer Biol..

[181] Kozomara A., Griffiths-Jones S. (2011). Mirbase: Integrating microRNA annotation and deep-sequencing data. Nucleic Acids Res..

[182] Iorio M.V., Ferracin M., Liu C.-G., Veronese A., Spizzo R., Sabbioni S., Magri E., Pedriali M., Fabbri M., Campiglio M. (2005). Microrna gene expression deregulation in human breast cancer. Cancer Res..

[183] Alvarez-Garcia I., Miska E.A. (2005). MicroRNA functions in animal development and human disease. Development (Cambridge, England).

[184] Krol J., Loedige I., Filipowicz W. (2010). The widespread regulation of microRNA biogenesis, function and decay. Nat. Rev. Genet..

[185] Metzler M., Wilda M., Busch K., Viehmann S., Borkhardt A. (2004). High expression of precursor microrna-155/BIC RNA in children with Burkitt lymphoma. Genes Chromosomes Cancer.

[186] Rana T.M. (2007). Illuminating the silence: Understanding the structure and function of small RNAs. Nat. Rev. Mol. Cell Biol..

[187] Lewis B.P., Burge C.B., Bartel D.P. (2005). Conserved seed pairing, often flanked by adenosines, indicates that thousands of human genes are microrna targets. Cell.

[188] Calin G.A., Sevignani C., Dumitru C.D., Hyslop T., Noch E., Yendamuri S., Shimizu M., Rattan S., Bullrich F., Negrini M. (2004). Human microrna genes are frequently located at fragile sites and genomic regions involved in cancers. Proc. Natl. Acad. Sci. USA.

[189] Calin G.A., Dumitru C.D., Shimizu M., Bichi R., Zupo S., Noch E., Aldler H., Rattan S., Keating M., Rai K. (2002). Frequent deletions and down-regulation of micro-RNA genes miR15 and miR16 at 13q14 in chronic lymphocytic leukemia. Proc. Natl. Acad. Sci. USA.

[190] Cimmino A., Calin G.A., Fabbri M., Iorio M.V., Ferracin M., Shimizu M., Wojcik S.E., Aqeilan R.I., Zupo S., Dono M. (2005). MiR-15 and miR-16 induce apoptosis by targeting BCL2. Proc. Natl. Acad. Sci. USA.

[191] Michael M.Z., O’Connor S.M., van Holst Pellekaan N.G., Young G.P., James R.J. (2003). Reduced accumulation of specific microRNAs in colorectal neoplasia11note: Susan M. O’connor and Nicholas G. Van Holst Pellekaan contributed equally to this work. Mol. Cancer Res..

[192] Chen C. (2005). Micrornas as oncogenes and tumor suppressors. N. Engl. J. Med..

[193] Zaravinos A. (2015). The regulatory role of microRNAs in EMT and cancer. J. Oncol..

[194] Gregory P.A., Bert A.G., Paterson E.L., Barry S.C., Tsykin A., Farshid G., Vadas M.A., Khew-Goodall Y., Goodall G.J. (2008). The miR-200 family and miR-205 regulate epithelial to mesenchymal transition by targeting Zeb1 and Sip1. Nat. Cell Biol..

[195] Park S.-M., Gaur A.B., Lengyel E., Peter M.E. (2008). The mir-200 family determines the epithelial phenotype of cancer cells by targeting the E-cadherin repressors Zeb1 and Zeb2. Genes Dev..

[196] Kumarswamy R., Mudduluru G., Ceppi P., Muppala S., Kozlowski M., Niklinski J., Papotti M., Allgayer H. (2012). MicroRNA-30A inhibits epithelial-to-mesenchymal transition by targeting snai1 and is downregulated in non-small cell lung cancer. Int. J. Cancer.

[197] Korpal M., Ell B.J., Buffa F.M., Ibrahim T., Blanco M.A., Celià-Terrassa T., Mercatali L., Khan Z., Goodarzi H., Hua Y. (2011). Direct targeting of Sec23a by miR-200s influences cancer cell secretome and promotes metastatic colonization. Nat. Med..

[198] Ma L., Young J., Prabhala H., Pan E., Mestdagh P., Muth D., Teruya-Feldstein J., Reinhardt F., Onder T.T., Valastyan S. (2010). MiR-9, a MYC/MYCN-activated microRNA, regulates E-cadherin and cancer metastasis. Nat. Cell Biol..

[199] Martello G., Rosato A., Ferrari F., Manfrin A., Cordenonsi M., Dupont S., Enzo E., Guzzardo V., Rondina M., Spruce T. (2010). A microrna targeting dicer for metastasis control. Cell.

[200] Kong W., Yang H., He L., Zhao J.-J., Coppola D., Dalton W.S., Cheng J.Q. (2008). MicroRNA-155 is regulated by the transforming growth factor β/Smad pathway and contributes to epithelial cell plasticity by targeting RhoA. Mol. Cell. Biol..

[201] Zhang J., Ma L. (2012). Microrna control of epithelial–mesenchymal transition and metastasis. Cancer Metastasis Rev..

[202] Milenkovic D., Deval C., Gouranton E., Landrier J.-F., Scalbert A., Morand C., Mazur A. (2012). Modulation of mirna expression by dietary polyphenols in apoE deficient mice: A new mechanism of the action of polyphenols. PLoS ONE.

[203] Li Y., VandenBoom T.G., Kong D., Wang Z., Ali S., Philip P.A., Sarkar F.H. (2009). Up-regulation of mir-200 and let-7 by natural agents leads to the reversal of epithelial-to-mesenchymal transition in gemcitabine-resistant pancreatic cancer cells. Cancer research.

[204] Hsieh T.C., Wu J.M. (2009). Targeting CWR22Rv1 prostate cancer cell proliferation and gene expression by combinations of the phytochemicals EGCG, genistein and quercetin. Anticancer Res..

[205] Saha A., Kuzuhara T., Echigo N., Suganuma M., Fujiki H. (2010). New role of (−)-epicatechin in enhancing the induction of growth inhibition and apoptosis in human lung cancer cells by curcumin. Cancer Prev. Res..

[206] Niedzwiecki A., Roomi M.W., Kalinovsky T., Rath M. (2016). Anticancer efficacy of polyphenols and their combinations. Nutrients.

